# Genome Sequence of an Efficient Indole-Degrading Bacterium, Cupriavidus sp. Strain IDO, with Potential Polyhydroxyalkanoate Production Applications

**DOI:** 10.1128/genomeA.00102-15

**Published:** 2015-03-12

**Authors:** Qiao Ma, Yuanyuan Qu, Zhaojing Zhang, Pengpeng Li, Hongzhi Tang

**Affiliations:** a Key Laboratory of Industrial Ecology and Environmental Engineering, School of Environmental Science and Technology, Dalian University of Technology, Dalian, China; b State Key Laboratory of Microbial Metabolism and School of Life Sciences and Biotechnology, Shanghai Jiao Tong University, Shanghai, China

## Abstract

Cupriavidus sp. strain IDO has been shown to efficiently transform indole, and the genus of Cupriavidus has been described as a promising cell factory for polyhydroxyalkanoate synthesis from low-cost wastes. Here, we report the draft genome sequence of strain IDO, which may provide useful genetic information on indole metabolism and polyhydroxyalkanoate production.

## GENOME ANNOUNCEMENT

Indole, a typical *N*-heterocyclic pollutant, is widespread in various environmental matrices. Since 1920s, the biodegradation of indole has been investigated, and general indole degradation pathways have been proposed (1, 2). However, the indole degradation efficiency of the reported strains was not satisfactory, and the related indole hydroxylase or dioxygenase that catalyzes the first step of indole oxidation was not identified (1, 3). Recently, a Gram-negative strain designated Cupriavidus sp. IDO, which utilizes indole as the sole carbon, nitrogen, and energy sources, was isolated from soil samples in Dalian, China. It was deposited at the China General Microbiological Culture Collection Center under accession no. CGMCC 9265. Strain IDO can remove 100 mg/liter indole within 15 h, which makes it the most efficient microorganism for indole degradation (Y. Qu, October 2014, China patent CN104099274A). The 16S rRNA gene sequence of IDO shares 99% similarity with that of Cupriavidus necator strains. Members of the genus Cupriavidus, especially C. necator, are recognized as amazing pollutant-degrading bacteria (4, 5). Studies also show that they are efficient platforms for the production of polyhydroxyalkanoates (PHAs), which are promising substitutes for conventional petrochemical plastics due to their biodegradable and biocompatible merits, as they use waste material as a substrate (6, 7). Here, the genome sequence of Cupriavidus sp. strain IDO may provide insights into its molecular mechanism and its indole degradation and PHA production.

The genome of strain IDO was sequenced using an Illumina HiSeq 2000 platform. The obtained reads were assembled into 206 large contigs using Velvet software (version 1.2.10) (8). Next, gene prediction and annotation were performed using the RAST autoannotation server (9). The genome sequence is 7,926,600 bp in length, with a G+C content of 64.58%. Strain IDO contains 68 RNAs and 536 subsystems in the genome. There are 7,457 predicted coding sequences (CDSs), accounting for 83.87% of the total sequences.

A rich set of 294 genes are related with the degradation of aromatic compounds, such as benzoate, *p*-hydroxybenzoate, phenol, toluene, biphenyl, and chloroaromatics. The gene redundancy for aromatic metabolism suggests that IDO is a versatile pollutant degrader. Many aromatic hydroxylation enzymes, such as dioxygenase, hydroxylase, cytochrome P450 enzymes, flavin monooxygenases, oxidases, and peroxidases, exist in the genome, all of which have been reported to be able to oxidize indole into hydroxyl indoles (10–12). At the same time, there are 161 CDSs and 192 CDSs annotated for antibiotic/toxic compound resistance and stress response, implying that strain IDO can be applied to various environmental conditions.

There are 126 CDSs annotated in total that are correlated with PHA synthesis. The three crucial players in PHA synthesis, i.e., polyhydroxyalkanoic acid synthase (5 CDSs), β-ketothiolase (15 CDSs), and acetoacetyl-coenzyme A (CoA) reductase (6 CDSs), are found in the genome. Among them, the gene *phaC1* of IDO shows 87% similarity with C. necator strain H16, a model strain for PHA synthesis (13). The genome sequence information obtained indicates that IDO should also be explored as a PHA-producing platform.

### Nucleotide sequence accession numbers.

This whole-genome shotgun project has been deposited at DDBJ/EMBL/GenBank under the accession no. JWMA00000000. The version described in this paper is the first version, JWMA01000000.
